# A global machine learning framework for classifying national CO2 emission levels: responsible and explainable artificial intelligence for sustainable climate governance

**DOI:** 10.3389/frai.2026.1943572

**Published:** 2026-10-16

**Authors:** Muhammad Ali Khawaja, Muhammad Farooq Shaikh

**Affiliations:** 1 Applied Economics Research Centre, University of Karachi, Karachi, Pakistan; 2 School of Engineering, University of Warwick, Coventry, United Kingdom

**Keywords:** carbon emissions, environment, explainable AI, governance, machine learning, taxation, trade

## Abstract

We investigate whether trade openness, governance, environmental taxation and selected macroeconomic indicators can classify the contemporaneous CO2 emission level of countries observed over 2000 to 2023, using data from the World Development Indicators and the OECD. CO2 emissions per capita are divided into three ordinal levels, low, medium and high, using tertile cut points estimated on the training data only within every validation split. Four classifiers, Random Forest, XGBoost, a support vector machine and a multinomial logistic regression, are tuned and compared alongside two models designed specifically for ordinal outcomes, an ordered logistic regression and an ordinal decomposition of XGBoost, under four validation designs: a country grouped holdout and country grouped 5 fold cross validation, in which no country appears in both the training and test sets; a chronological holdout; a 1 year ahead forecasting task; and a random stratified country year split, retained only as a reference point. Under country grouped cross validation, no classifier achieves a decisively higher accuracy than the others: nested cross validation gives a mean accuracy of 0.644 for XGBoost, 0.648 for Random Forest, 0.671 for the support vector machine, 0.674 for the multinomial logit, 0.676 for the ordered logit and 0.637 for the ordinal XGBoost model, with overlapping confidence intervals throughout. This is markedly lower than the 0.91 accuracy obtained under a random country year split with the same tuned pipeline, a gap of 12.5 percentage points for XGBoost, confirming that a substantial part of the high accuracy obtainable under random splitting reflects the model recognizing previously seen country profiles rather than generalizing to unseen countries. Accuracy falls less sharply under the chronological holdout and the 1 year ahead forecasting task, in the region of 0.65 to 0.74, indicating that the classifiers generalize better across time within a country than across countries. Permutation importance, SHAP and LIME consistently identify government effectiveness, urban population growth and environmental tax revenue as the features most strongly associated with model predictions, with a moderate contribution from trade openness and the smallest from GDP growth, a ranking that is stable across importance methods, learning algorithms and cross validation folds. These variables are predictors of the model's output, not estimated causal determinants of emissions, and the results are best read as evidence for climate risk monitoring and further policy evaluation rather than as a causal policy assessment.

## Introduction

In the twenty first century, climate change has become one of the top economic and environmental concerns. Due to rapid environmental degradation, global warming and extreme climatic events, sea level rise, biodiversity loss and the pressure on food and water resources, the study of carbon dioxide emissions is of high importance for researchers and policy makers. CO2 is particularly important to climate research as it is the main greenhouse gas forcing, and World Meteorological Organization observations indicate that the concentration of greenhouse gases in the atmosphere is at high levels, with CO2 concentrations currently higher than at any time in at least two million years (WMO, 2023). Due to its close relationship with various aspects of production, energy consumption, urbanization, trade, investment and institutional capacity, this makes CO2 emissions an economic development issue as well as an environmental issue.

There is a growing emphasis on CO2 emissions reductions in all significant international climate agreements. The Kyoto Protocol was one of the first legally binding international climate agreements that set targets for greenhouse gas emission reductions to be achieved by the industrialized nations within the United Nations climate framework (United Nations, 1997). Subsequently, the Paris Agreement was adopted as the main global climate agreement, and established the goal of the increase in the global average temperature being well below 2 degrees Celsius above the pre industrial level, and to strive to limit the warming to 1.5 degrees Celsius (United Nations, 2015). The agreements demonstrate that climate change mitigation is not a purely scientific issue, but also an issue of international cooperation, national policy design and economic restructuring.

The importance of CO2 patterns between countries has been further enhanced by the need for net zero emissions. Net zero is a balancing of emissions at the lowest level possible, close to zero, with the remaining emissions removed or permanently stored. The Intergovernmental Panel on Climate Change emphasizes that significant and rapid cuts in greenhouse gas emissions are essential to keep global warming within limits; reaching net zero CO2 emissions is a key element for achieving global temperature stabilization (Lee et al., 2023). Similarly, the International Energy Agency states that the road toward achieving net zero by 2050 is tight and demands swift and vast scale up of clean and efficient energy technologies (International Energy Agency, 2021).

Even with the global commitments, the world is still off track as indicated by recent assessments. The United Nations Environment Programme Emissions Gap Report estimates that current policy trends are not enough to reach the 1.5 degree target and recommends increased efforts on mitigation (United Nations Environment Programme, 2024). This growing disparity between climate ambition and actual emissions outcomes underscores the need for empirical studies that identify the features which best classify countries by their emission level. Evidence of this kind can help inform more effective policy prioritization among countries that experience varying levels of development, trade exposure, governance quality, levels of urbanization and environmental tax capacity.

This study seeks to investigate whether trade openness, governance indicators, environmental taxation and selected macroeconomic variables can classify countries into CO2 emission levels, and whether that classification generalizes to countries and periods the model has not seen. Existing machine learning studies of CO2 emissions either treat emissions as a continuous outcome predicted from energy and economic variables in a small number of large emitters or a single country (Ahmed et al., 2022; Qin and Gong, 2022; Yu et al., 2024; Li et al., 2021), or classify countries on other sustainability, governance or innovation indices without treating emissions themselves as an ordinal, panel structured outcome and without testing generalization across countries or time (Celik et al., 2026a, 2025; Celik and Koroglu, 2026; Begum and Al Mobin, 2025). This study addresses that gap in three ways: first, by classifying CO2 emission levels for a broad 135 country sample using trade, governance and environmental tax indicators alongside macroeconomic controls; second, by testing whether the resulting classifiers generalize across countries and across time, not only across randomly split country year observations; and third, by establishing, through permutation importance, SHAP and LIME jointly, which of these predictors carry a stable association with the classification and in which direction. The paper makes recommendations on incorporating trade policy, institutional quality and environmental taxation into climate monitoring and emissions classification frameworks, framed throughout as predictive association rather than causal evidence.

### Top CO2 emitters

The average CO2 emission levels of the top 10 emitters in the selected sample of 135 countries are displayed in Figure 1, based on CO2 emissions during 2021 to 2023. A clear pattern is seen. The top countries are either highly developed or with high level of resource wealth or oil and gas rich economies. The top two countries are Brunei Darussalam and Trinidad and Tobago, which is unsurprising given both economies' close ties to oil and gas exploration and production. Other relatively developed, high income countries in the sample, such as Canada, the United States, Australia, Luxembourg, Singapore and Estonia, also show high energy consumption and transport demand, with industrial or service sector activity that contributes to higher emissions per capita.

**Figure 1:**
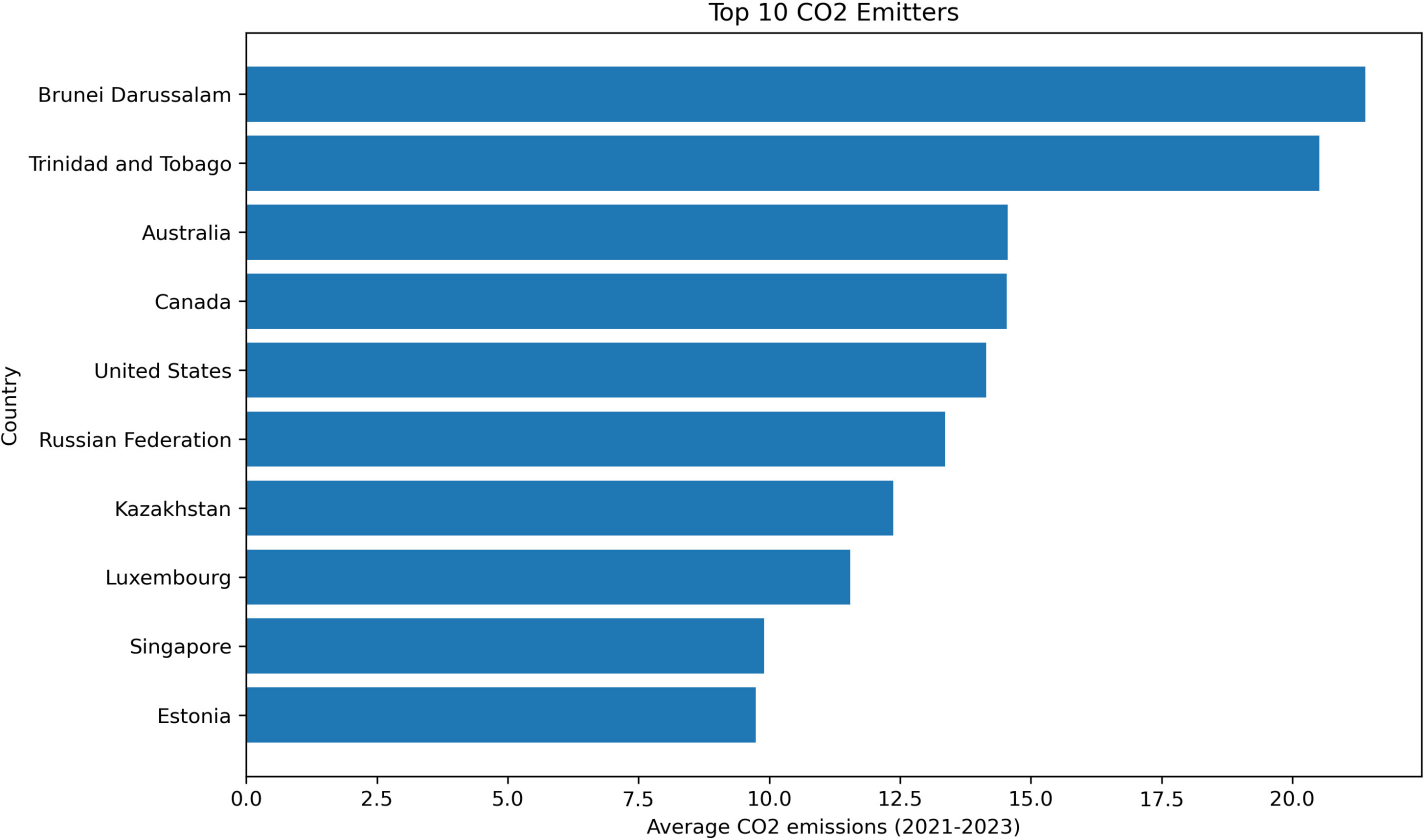
Average per capita CO2 emissions of the top 10 emitting countries in the study sample, 2021 to 2023. Source: Authors' estimation based on World Development Indicators and OECD data.

The influence of fossil fuel production, heavy industry and resource based economic structures on emission levels is also evident in the presence of the Russian Federation and Kazakhstan. High CO2 emission categories are therefore correlated not only with population size but also with energy structure, dependence on fossil fuels, industrial activity and income.

Building on this, the high emitter pattern in the sample is also broadly in line with global evidence on emissions concentrations and fossil fuel dependency. The Global Carbon Budget report shows that global fossil CO2 emissions reached a new peak of 37.4 gigatonnes in 2024, driven primarily by continued fossil fuel use, but with emissions growth slowing in some advanced economies (Friedlingstein et al., 2025).

The Global Carbon Budget additionally indicates that fossil CO2 emissions are still rising at a world scale and offers national fossil carbon emissions datasets, illustrating that the emissions of countries are significantly influenced by their use of fuels, their industrial processes and their economic structures based on resources. This is applicable to countries in the sample that have oil, gas, mining or other resource based sectors that are included in the economy, including Brunei Darussalam, Trinidad and Tobago, the Russian Federation, Kazakhstan, Australia and Canada.

Moreover, the United Nations Environment Programme Emissions Gap Report highlights the lack of mitigation ambition by many high emitting economies to meet the Paris Agreement temperature targets, highlighting the need to intensify policy action on measures to transition the energy sector, tax environmental pollution, decarbonize industry and strengthen governance (United Nations Environment Programme, 2024).

## Literature review

In the literature, a strong trend in the use of machine learning techniques for the analysis of CO2 emissions is observed in recent years. The machine learning approach can identify non-linearities and complex patterns in predictions which are not possible with traditional econometric models.

A study of influencing factors using machine learning methods in the context of carbon emissions in India and China was conducted by Ahmed et al. (2022). The study is valuable because it is based on two of the largest developing economies in the world, both of which have experienced significant environmental strain due to population growth, energy demand and industrialization. The five key explanatory variables that the authors used were energy consumption, financial development, GDP, population and renewable energy. They concluded that energy use was the biggest factor affecting CO2 emissions, whereas renewables were the least significant. The research also suggested that emissions could be reduced by raising the use of renewables and decreasing that of coal, without harming economic development. The relevance of this work is that it demonstrates the possibilities of using machine learning to determine the relative importance of economic and energy related variables in explaining emission patterns.

Another key contribution was made by Qin and Gong (2022), who used machine learning techniques to estimate CO2 emissions and their driving factors in China. They applied provincial CO2 emissions data and nighttime light data to calculate city level emissions for Chinese prefecture level cities from 2000 to 2019. They then used machine learning models, such as decision tree and random forest, to determine what factors are most important in driving emissions. They found that emissions are higher in the east, where the economies are more developed, and in resource dependent cities. The feature importance from the random forest showed that GDP, financial general budget revenue and foreign investment were the key features that predict CO2 emissions. This study is highly relevant as it shows the usefulness of random forest for ranking economic drivers of emissions, which is directly related to the approach of the present study.

Building on the growing body of machine learning literature, Ayimadu et al. (2024) investigated the determinants of carbon emissions across 48 African countries over the period 2000 to 2022 using a range of machine learning techniques. This study is relevant since African countries must support economic growth while keeping the environment sustainable. In addition to ordinary least squares regression, the authors employed several machine learning models such as random forest, extra trees, histogram gradient boosting and decision tree models. From their findings, they concluded that CO2 emissions rose in Africa with economic growth and energy use. They also discovered that natural resource rents had a positive effect on environmental unsustainability, but that this weakened over the second decade of the sample. This study is significant in that it demonstrates the use of machine learning in multi country panel settings and its capability to provide policy suggestions to developing regions.

Yu et al. (2024) analyzed carbon emissions over a 10 year period, 2011 to 2020, in 254 Chinese cities and compared six machine learning algorithms to traditional econometric models. Their study is of special value because, in addition to making predictions, it detects non-linear relationships between emissions and their drivers. They considered internal and external economic variables, policy uncertainty, government interference, digital finance and energy usage. Their results indicated that energy consumption continued to be the main source of increasing carbon emissions, while government interventions and digital finance contributed to reducing carbon emissions. The study also revealed that external economic and policy uncertainty could influence emissions, with economic policy uncertainty having the greatest impact. This study further demonstrates that machine learning is an effective tool for understanding the complex relationship between institutional, economic and policy variables and emissions.

Selmeya et al. (2025) used several machine learning algorithms to analyze carbon emissions in the top 10 carbon emitting countries between 1990 and 2023: random forest, logistic regression, support vector machines, K nearest neighbors and gradient boosting. This study is particularly valuable because it deals with a large proportion of countries that contribute to global emissions. The authors concluded that random forest performed close to perfect on multiple metrics of prediction. They also found that primary energy use had the greatest effect on carbon emissions, with population size and GDP as the second and third largest factors, respectively, whereas trade openness, urbanization and renewable energy use had relatively smaller effects. This study is relevant since it provides support for the use of random forest in emissions forecasting and shows the role of feature importance analysis in determining the strongest model for predicting CO2 emissions.

Li et al. (2024) turned attention away from national emissions and toward the transport sector, estimating emissions of CO2 from light duty diesel trucks using machine learning. They studied real world vehicle emissions using data collected from a portable emission measurement system and GPS data. The authors built a deep learning model with a long short term memory structure and introduced vehicle specific power, velocity, road slope and acceleration as input variables to predict CO2 emissions. They found that emission rates were influenced significantly by speed, road slope and acceleration, with acceleration above a certain threshold generating high CO2 emissions. This model performed very well in terms of predictive performance, with R squared values of 0.986 to 0.990. This paper demonstrates that machine learning can also be effective for sector specific emissions prediction, with clear applications to transport policy and fleet management.

Lastly, Li et al. (2021) analyzed the drivers of CO2 emissions for China using various machine learning algorithms. They analyzed economic growth, the industrial system, urbanization, research and development investment, foreign investment and energy use between 2000 and 2018. Model training was performed using data from the 15 years to 2015, and predictions were made for the following 3 years, 2016 to 2018. They found that the k nearest neighbors model worked best for their dataset. The study also revealed a non-linear relationship between industrialization, urbanization and CO2 emissions, such that at different stages and levels of development, CO2 emissions can either rise or fall. It concluded that research and development investment had a negative impact on emissions, particularly in combination with green technology and foreign investment. This study is significant as it provides an example of how machine learning can inform policy by identifying optimal ranges and non-linear emission responses.

A parallel and more recent strand applies machine learning and explainable artificial intelligence to cross country classification problems outside the emissions literature narrowly defined, and is methodologically closer to the present design because it treats country year or country level observations as the unit of classification and uses SHAP based explanation jointly with clustering or supervised classifiers. Begum and Al Mobin (2025) forecast CO2 emissions to 2030 for the 11 largest emitters against Paris Agreement targets using six machine learning models with sequential feature selection. Celik et al. (2025) cluster 166 countries on the 2025 Sustainable Development Goal Index and classify cluster membership with random forest, support vector machines and neural networks. Celik (2026) applies machine learning to classify countries by economic freedom performance, and Celik and Koroglu (2026) combine clustering with SHAP to analyze global gender inequality patterns across 146 countries. Celik et al. (2026a) use principal component analysis and explainable machine learning to identify non-linear regimes in global innovation systems, Celik and Sasmaz (2026) map latent environmental performance regimes with a spatial, explainable machine learning approach across 180 countries, and Celik et al. (2026b) apply similar methods to global budget transparency. These studies confirm that tree based ensembles paired with SHAP are now an established approach for cross country structural classification, but none of them evaluates generalization to countries or periods withheld from training, which is the gap this study's validation design is built to close.

Overall, the literature indicates that machine learning is a significant instrument in CO2 emissions studies. Most of the previous research has been based on China, India, Africa, transport emissions or the top emitting countries. Most of these studies indicate that energy consumption, GDP, institutional and policy factors, industrialization, population and urbanization are significant predictors of emissions. Fewer studies specifically classify countries by CO2 emission level based on the combination of trade openness, governance and environmental taxes, and fewer still test whether such classifiers generalize beyond the countries and periods used to train them. The present study addresses this gap by classifying CO2 emission levels, low, medium and high, using different machine learning algorithms for 135 countries, determining the relative importance of trade, governance and environmental taxation alongside other macroeconomic variables, and evaluating generalization under country grouped, chronological and forecasting validation designs rather than a random country year split alone.

## Materials and methods

### Data and machine learning models

The study employs a country level panel dataset for 135 countries around the world during the period 2000 to 2023 for the analysis. The number of countries included in the study was determined by data availability within the OECD database. A list of countries included in the sample is attached in the Appendix. The World Development Indicators database was used for the collection of data, apart from the environmental tax variable, which was drawn from the OECD database. The aim of a multi country dataset is to reflect variations in the economies of the countries covered, in their trade exposure, in their governance quality, in their environmental taxation, in their labor market conditions, in their capital formation, in their urbanization and in their CO2 emissions in a large international sample.

The dependent variable of the study is CO2 emissions per capita, which represents each country's level of carbon emissions. The study treats CO2 emissions as an ordinal categorical variable rather than a continuous variable, consistent with the paper's goal of classifying countries into CO2 emission levels and assessing the ability of selected economic, governance, trade and environmental policy variables to classify these emission levels. See Table 1 for variables' description.

**Table 1:** Description of the variables, measurement and data sources used in the machine learning framework.

Variable	Code	Source
CO2 emissions per capita	CO2	World Development Indicators
Trade (% of GDP)	Trade	World Development Indicators
GDP growth (annual %)	GDPGrowth	World Development Indicators
Government effectiveness (score 0–100)	GOV	World Development Indicators/Worldwide Governance Indicators
Rule of law (score 0–100)	RULE	World Development Indicators/Worldwide Governance Indicators
Gross fixed capital formation (% of GDP)	GFCF	World Development Indicators
Urban population growth (annual %)	UrbanGrowth	World Development Indicators
Labor force participation rate (%)	LFPR	World Development Indicators
Environmental taxes (revenue % of GDP)	ET	OECD

### Construction of the ordinal target and prevention of information leakage

CO2 emissions per capita are divided into three ordinal levels using tertile cut points. Because the sample is a country year panel, computing these cut points on the pooled sample before splitting the data would let information from the test observations shape the target definition. To avoid this, the tertile cut points are re estimated on the training portion of each validation split only and then applied unchanged to the corresponding validation or test observations. Under the primary, country grouped split, the resulting cut points are 1.32 and 5.04 tons of CO2 per capita; for reference, the cut points obtained from the pooled sample, including test observations, are 1.35 and 5.00 tons per capita, close to but not identical with the training only values, illustrating the leakage the training only procedure is designed to avoid. The dependent variable is coded ascendingly, 0 for low, 1 for medium and 2 for high.

Because this ordering is substantive, the four principal classifiers are supplemented with two models designed specifically for an ordinal outcome, an ordered, proportional odds logistic regression, and an ordinal decomposition of XGBoost following Frank and Hall (2001), which reduces the three class problem to two nested binary classifiers, the probability that the emission level exceeds low, and the probability that it exceeds medium. Because Random Forest, XGBoost, the support vector machine and the multinomial logit do not otherwise impose an ordinal loss on the three categories, every model, ordinal and non-ordinal alike, is evaluated with quadratic weighted and linear weighted Cohen's kappa and mean absolute error on the class labels, in addition to accuracy and macro F1, so that adjacent category errors and two category, severe, errors are scored differently rather than treated as equally wrong. Refer to Figure 2 for complete analytical workflow.

**Figure 2:**
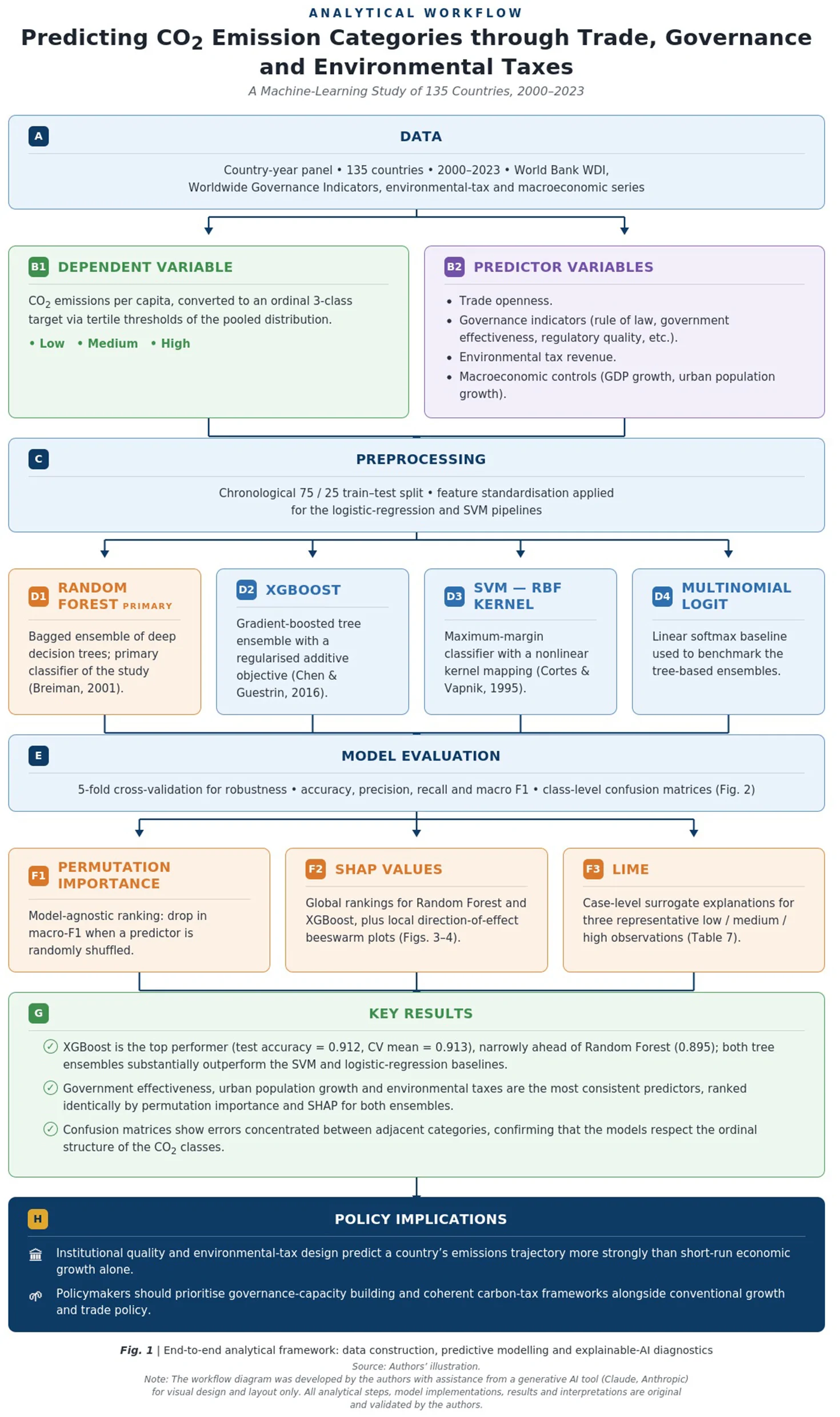
Analytical workflow of the proposed machine learning framework for classifying national CO2 emission levels, including the country grouped, chronological and random holdout splits compared in this study. The workflow diagram was developed by the authors with assistance from a generative artificial intelligence tool, Claude, Anthropic, for visual design and layout only. The analytical framework, methodology, model implementation, results and scientific interpretation were conceived, verified and validated by the authors.

### Validation design

The dataset comprises repeated observations of the same countries across 24 years. A validation design that allocates country year observations to training and test sets at random allows the same country to appear in both sets in different years. Because national institutional quality, urbanization, trade structure and emissions are highly persistent from 1 year to the next, such a design risks measuring a model's ability to recognize previously seen country profiles rather than its ability to generalize to unseen countries or future periods. This revision reports four validation designs side by side, summarized in Table 2.

**Table 2:** Classification performance of six machine learning classifiers under four validation designs.

Design	Model	Accuracy	Balanced Acc.	Macro F1	QWK	MAE	Severe error %	95% CI (Acc.)
Country grouped holdout	XGBoost	0.624	0.629	0.616	0.670	0.393	1.6	0.507–0.727
	Random forest	0.629	0.634	0.622	0.667	0.390	1.9	0.510–0.729
	SVM (RBF)	0.650	0.655	0.645	0.697	0.361	1.1	0.531–0.753
	Multinomial logit	0.622	0.629	0.617	0.686	0.388	1.0	0.503–0.728
	Ordered logit	0.631	0.637	0.626	0.660	0.394	2.5	0.514–0.729
	Ordinal XGBoost	0.640	0.646	0.632	0.676	0.380	2.0	0.525–0.744
Chronological holdout	XGBoost	0.710	0.730	0.718	0.735	0.295	0.5	0.641–0.770
	Random forest	0.740	0.763	0.748	0.763	0.265	0.5	0.674–0.798
	SVM (RBF)	0.672	0.695	0.682	0.713	0.330	0.1	0.599–0.746
	Multinomial logit	0.646	0.686	0.655	0.698	0.359	0.5	0.572–0.718
	Ordered logit	0.658	0.685	0.670	0.707	0.342	0.0	0.588–0.726
	Ordinal XGBoost	0.716	0.740	0.724	0.741	0.290	0.6	0.650–0.777
One year ahead forecast	XGBoost	0.710	0.730	0.717	0.730	0.298	0.8	0.642–0.771
	Random Forest	0.702	0.722	0.710	0.720	0.307	0.9	0.631–0.768
	SVM (RBF)	0.672	0.694	0.682	0.710	0.331	0.3	0.595–0.747
	Multinomial logit	0.651	0.687	0.660	0.694	0.356	0.8	0.578–0.726
	Ordered logit	0.656	0.685	0.668	0.705	0.345	0.1	0.584–0.726
	Ordinal XGBoost	0.700	0.724	0.708	0.725	0.308	0.8	0.635–0.761
Random split (reference)	XGBoost	0.749	0.749	0.751	0.788	0.260	0.9	0.687–0.805
	Random forest	0.766	0.766	0.769	0.799	0.242	0.8	0.705–0.820
	SVM (RBF)	0.726	0.726	0.726	0.770	0.284	1.0	0.664–0.785
	Multinomial logit	0.695	0.694	0.689	0.745	0.321	1.5	0.626–0.756
	Ordered logit	0.690	0.689	0.690	0.726	0.328	1.8	0.627–0.748
	Ordinal XGBoost	0.768	0.768	0.769	0.798	0.243	1.1	0.712–0.821

QWK is quadratic weighted Cohen's kappa. Severe error % is the share of test observations misclassified by two categories. Confidence intervals are 95 percent country cluster bootstrap intervals with 500 replicates. Country grouped test set, *n* = 792 from 33 countries, class distribution 253 low, 280 medium, 259 high. Chronological and 1 year ahead test sets, *n* = 786 each; random split test set, *n* = 786.

The first and primary design is a country grouped holdout and country grouped 5 fold cross validation. Countries, not country year observations, are the sampling unit: each country's full run of years falls entirely in the training set or entirely in the test set, so no model is ever evaluated on a country it has seen during training. The 75 to 25 holdout partition and the five cross validation folds are chosen, among repeated random country level allocations, to keep each split's emission level composition close to balanced. The second design is a chronological holdout, in which all country years through 2017 are used for training and 2018 to 2023 for testing, so the model is evaluated on genuinely future observations for countries it may have seen in earlier years. The third design is a 1 year ahead forecast, in which predictors measured in year t are used to predict the emission level in year t plus 1, with a chronological split on the target year, training on target years through 2017 and testing on 2018 to 2023. The fourth design is a random stratified country year split, identical in spirit to the design used in the original submission, retained only as a reference point to quantify how much of a previously reported high accuracy can reflect within country persistence rather than genuine generalization.

All tuned models in every design, including the random split reference, are selected by hyperparameter search using a country grouped inner cross validation, so that the comparison across designs isolates the effect of the outer validation scheme itself rather than confounding it with different degrees of overfitting from an untuned model. A randomized search over 20 candidate configurations is used for XGBoost, Random Forest, the support vector machine, the multinomial logit and the ordinal XGBoost model, evaluated with a 3 fold country grouped inner cross validation nested inside each training set and optimizing balanced accuracy; the outer, held out test set of each design is touched only once, after the inner search is complete. Selected hyperparameters for the primary, country grouped design are reported in the Results, Table 3. Uncertainty around the point estimates is quantified in two ways: a country cluster bootstrap, resampling whole countries with replacement across 500 replicates, for 95 percent confidence intervals around accuracy and quadratic weighted kappa, reported alongside accuracy in Table 2; and repeated five by five country grouped cross validation for the stability of the mean accuracy across independent fold assignments, discussed in the Results.

**Table 3:** Selected hyperparameters under the primary, country grouped design.

Model	Selected hyperparameters
XGBoost	574 trees, learning rate 0.010, max depth 2, min child weight 1, subsample 0.656, column subsample 0.845, L2 penalty 1.674
Random forest	539 trees, max depth 5, min samples per leaf 2, max features square root
Ordinal XGBoost	472 trees per binary sub model, learning rate 0.014, max depth 4, min child weight 8, subsample 0.840, column subsample 0.778

The support vector machine, multinomial logit and ordered logit were also tuned by the same randomized search procedure; their selected hyperparameters are available from the corresponding author on request.

### The Random Forest classifier

The Random Forest classifier is one of the primary models in the study, used to classify the ordinal CO2 emission level of each country year observation (Kalange et al., 2022). Random Forest is a supervised machine learning method based on many trees. Each tree classifies the dependent variable by repeatedly splitting the data according to the independent variables. The final prediction is obtained by combining the predictions of all trees through majority voting.

The dependent variable in this study is the ordinal CO2 emissions category, Yit, belonging to the set of zero, one and two, where Yit represents the CO2 emissions category of country i in year t, with 0 defined as low CO2 emissions, 1 as medium and 2 as high. The set of independent variables Xit is defined as Trade, GDP growth, government effectiveness, rule of law, gross fixed capital formation, urban population growth, labor force participation and environmental taxes, all measured for country i in year t.

The prediction model may be written as Yit equals f of Xit, where f represents the Random Forest classification function, defined as the mode of the predictions of the individual trees T1 through TB applied to Xit, with B the total number of trees in the forest. Each tree is trained on a random sample of the data and uses a random subset of variables at each split. This allows the model to capture non-linear relationships and interactions among variables.

### Comparison models: XGBoost, ordinal models, support vector machine, and logistic regression

To assess the robustness of the results and to compare predictive performance across different classes of learning algorithms, five additional classifiers are estimated: an Extreme Gradient Boosting classifier, an ordinal decomposition of XGBoost, an ordered logistic regression, a support vector machine and a multinomial logistic regression.

The XGBoost classifier serves as a second tree based ensemble (Fouladvand et al., 2023). While Random Forest is a bagging ensemble in which many deep trees are grown independently on bootstrap samples and combined through majority voting, XGBoost is a boosting ensemble in which shallow trees are grown sequentially, with each new tree fitted to the errors of the ensemble built so far. The model is estimated in an additive, stage wise manner, minimizing a regularized objective function composed of a differentiable loss term, the multiclass softmax loss in this study, and a complexity penalty on the number of leaves and the magnitude of the leaf weights in each tree, which protects against overfitting. Class imbalance is addressed through inverse frequency sample weights, analogous to the balanced class weights used in the Random Forest.

The ordinal decomposition of XGBoost, following Frank and Hall (2001), fits two binary XGBoost classifiers on the same features, one for the probability that the emission level exceeds low and one for the probability that it exceeds medium, and combines them into a three category probability distribution that respects the ordering of the classes by construction. The ordered logistic regression models the cumulative probability that an observation falls at or below a given category as a function of a single linear index and a set of ordered thresholds, so that, unlike the multinomial logit, it treats the three categories as ordered points on a single underlying scale rather than as unordered alternatives.

The multinomial logistic regression serves as the linear, non-ordinal baseline. It models the probability that observation i belongs to emission category k through the softmax function, with a separate coefficient vector for each category. Because the model imposes linear decision boundaries in the feature space, the comparison between the logistic regression and the tree based ensembles provides a measure of the extent to which the relationship between the predictors and CO2 emission categories is non-linear.

The support vector machine classifier separates classes by constructing the hyperplane that maximizes the margin, the distance between the decision boundary and the nearest training observations (Cortes and Vapnik, 1995). To accommodate non-linear class boundaries, the study employs the radial basis function kernel, which implicitly maps the features into a higher dimensional space in which a linear separating hyperplane corresponds to a non-linear boundary in the original feature space. Multiclass classification is handled through the one vs. rest scheme, and balanced class weights are applied, consistent with the tree based models.

Because the support vector machine, the multinomial logit and the ordered logit are sensitive to the scale of the input variables, their preprocessing pipelines include feature standardization, zero mean and unit variance, in addition to median imputation fitted on the training fold only. The tree based models do not require scaling, as decision tree splits are invariant to monotonic transformations of the features. All six classifiers are evaluated under each of the four validation designs described above, with hyperparameters tuned separately within each design's training data, so that differences in performance are attributable to the learning algorithms and the validation design rather than to inconsistent preprocessing.

### Model evaluation

Classification related measures are used to evaluate model performance. Because the dependent variable is categorical, the traditional regression R squared is not used. Rather, each classifier generates accuracy, balanced accuracy, precision, recall, F1 score and a confusion matrix. Because the dependent variable is ordinal, accuracy and macro F1 alone treat a high emission observation predicted as low and one predicted as medium as equally wrong, even though the first error is substantively more severe. Every model is therefore additionally evaluated with quadratic weighted and linear weighted Cohen's kappa, mean absolute error computed on the 0, 1, 2 class labels, and the share of predictions that are off by two categories, a severe error. Predictive uncertainty is summarized with the multiclass Brier score and the mean Shannon entropy of the predicted class probabilities, and classification performance is additionally broken down by World Bank region and by OECD membership to assess whether accuracy is concentrated in the better represented, higher income part of the sample given the OECD sourced environmental tax variable.

### Feature importance and model explainability

The study uses permutation feature importance to determine the most significant variables involved in classification. This approach quantifies how much a model's performance suffers when one independent variable is randomized while all other independent variables are held constant. If shuffling a variable results in a large loss of performance, that variable is regarded as an important predictor. Permutation importance is used to rank the variables in order of their contribution to classifying CO2 emission levels, including trade openness, governance, environmental taxes and the remaining predictors. The results are interpreted as predictive, not causal, importance.

To complement permutation feature importance and enhance the interpretability of the classifiers, the study employs two *post-hoc* explainable artificial intelligence techniques, Shapley Additive Explanations, SHAP, and Local Interpretable Model agnostic Explanations, LIME. SHAP is grounded in cooperative game theory and attributes each prediction to the model's input variables using Shapley values (Lundberg and Lee, 2017). Because the main classifiers in this study are tree based, the computationally efficient TreeSHAP algorithm is used (Lundberg et al., 2020). SHAP is applied both globally, by ranking features according to their mean absolute SHAP values across the test set and each CO2 category, and locally, through summary plots that reveal the direction of each variable's effect.

LIME explains individual predictions by approximating the classifier locally with an interpretable surrogate model (Ribeiro et al., 2016). In this study, LIME is used to explain the classification of a sample of correctly classified and misclassified observations drawn from each emission category, rather than a single hand picked case per category. Up to five correctly classified and up to five misclassified observations are drawn at random, with a fixed random seed, from each actual emission level in the country grouped test set, giving 30 cases in total, and both the individual rules and the class level mean absolute weight by feature across all sampled cases are reported, to avoid the appearance that the explanation rests on a small number of selectively chosen observations.

Because several governance and macroeconomic predictors are plausibly correlated, government effectiveness and rule of law in particular, Spearman rank correlations and variance inflation factors are computed for all eight predictors before the importance rankings are interpreted. To move beyond the marginal, one variable at a time view that SHAP summary plots and permutation importance provide, the analysis also reports accumulated local effects and partial dependence plots for the three highest ranked predictors, and SHAP interaction values for the XGBoost model, to identify which pairs of predictors contribute jointly to the classification beyond their individual effects. The stability of the importance ranking is assessed directly, by recomputing permutation importance within each fold of the country grouped cross validation and reporting the rank correlation of the resulting orderings across folds, rather than inferred indirectly from cross method agreement alone.

The methods are complementary: SHAP provides theoretically consistent global and local attributions, LIME offers intuitive case specific explanations, and permutation importance provides a model agnostic global ranking. Together they allow the study to verify whether the ranking of predictors is robust across interpretability techniques, across learning algorithms and across cross validation folds, and to identify the direction of each predictor's association with CO2 emission categories.

### Ethical considerations and responsible artificial intelligence

Given the growing use of artificial intelligence in climate research, it is important that the predictive models used are transparent, interpretable and trustworthy. This research combines permutation feature importance, SHAP and LIME to offer both local and global explanations of model behavior, so that the framework is not simply a black box classifier but gives policymakers insight into how governance quality, environmental taxation, trade openness and macroeconomic indicators are associated with countries' CO2 emission classifications (Randeniya et al., 2026; Mishra et al., 2026).

While machine learning tools offer important predictive functions, they can also reflect historical socioeconomic, institutional and governance biases in the data used in their creation. The associations found in this study are correlative, not causal, and the purpose of the proposed framework is to assist policy development, not to supersede expert judgement. Economic analysis, domain expertise and policy context, together with the evidence generated here, should continue to inform climate related decisions, and meaningful human oversight throughout the decision making process helps ensure fair, accountable and responsible use of artificial intelligence while minimizing the potential for unintended policy outcomes (Amore and Gentili, 2026).

Consistent with a responsible artificial intelligence framing, this study reports where the design is most exposed to bias, leakage and unmodeled uncertainty, rather than asserting these properties in general terms. Data representativeness is assessed directly through the region and OECD membership breakdown reported in the Results, since the OECD source for environmental tax revenue constrains coverage of some developing regions. Missingness is reported by variable and by country, and complete case analysis is compared against iterative imputation as a robustness check, to show that the main findings are not an artifact of how missing values are handled. Potential information leakage is addressed structurally rather than by assertion: tertile thresholds, imputation and feature scaling are all fitted within training folds only, and the country grouped, chronological and 1 year ahead validation designs directly test whether the model's apparent performance depends on having seen a country or a later period during training. Model uncertainty is reported through country cluster bootstrap confidence intervals on the headline accuracy and kappa statistics, and through the mean predictive entropy of each model's class probabilities, which is highest for the medium emission category and for the lower coverage regions identified in the Results, a signal that the model's explanations should be weighted less heavily for those observations.

In addition to classification, the framework aims to facilitate sustainable climate governance with evidence that is transparent and interpretable for policymaking. By identifying governance quality, urban population growth and environmental taxation as the predictors most strongly and consistently associated with a country's CO2 emission level, and by reporting honestly how much of that predictive signal survives once the validation design accounts for the panel structure of the data, the framework is intended to support climate monitoring and further policy evaluation rather than to assert a causal policy conclusion on its own (Gavali et al., 2026).

## Results

### Sample and missingness

The panel comprises 135 countries observed from 2000 to 2023, a maximum of 3,240 country year observations. CO2 emissions data were unavailable for the full sample period for four countries, Liechtenstein, Montenegro, Nauru and Serbia, which are listed in the Appendix as part of the original sampling frame but excluded from the estimation sample; after removing the 96 country year rows with a missing or non-positive CO2 value, the estimation sample comprises 3,144 country year observations from 131 countries. Missingness among the eight predictors ranges from 0.0 percent for urban population growth to 12.0 percent for environmental tax revenue, with an overall predictor missingness of 5.9 percent; trade openness, 9.6 percent, and gross fixed capital formation, 11.9 percent, are the next most affected variables. Imputation, feature scaling and tertile threshold estimation are fitted within training folds only in every validation design, and are never re fitted on validation or test data.

### Validation design comparison

Table 2 reports classification performance for all six classifiers under the four validation designs described in Materials and Methods. Under the random stratified country year split, kept only as a reference to the design used in the original submission, XGBoost achieves a test accuracy of 0.749 and a quadratic weighted kappa of 0.788, with Random Forest close behind at 0.766 accuracy. Under the primary, country grouped design, in which no country appears in both the training and test sets, accuracy falls markedly for every classifier: 0.624 for XGBoost, a drop of 12.5 percentage points, 0.629 for Random Forest, 0.650 for the support vector machine, 0.622 for the multinomial logit, 0.631 for the ordered logit and 0.640 for the ordinal XGBoost model. This confirms that a substantial part of the accuracy obtainable under a randomly stratified country year split reflects the model recognizing previously observed country profiles rather than generalizing to unseen countries.

Accuracy falls less sharply under the chronological holdout, in which all models are trained on data through 2017 and tested on 2018 to 2023, and under the 1 year ahead forecasting design, in which predictors from year t are used to classify the emission level in year t plus 1. Random Forest reaches 0.740 accuracy under the chronological holdout and the ordinal XGBoost model reaches 0.716; under the 1 year ahead forecast, XGBoost reaches 0.710 and the ordinal XGBoost model 0.700. Both of these designs still involve some overlap of countries between the training and test periods, so this pattern indicates that the classifiers generalize noticeably better across time within a country than across previously unseen countries, and that the country grouped design is the more conservative and, for a classification framework intended to support monitoring of countries in general, the more relevant test of generalization.

A country cluster bootstrap comparison of XGBoost against each other classifier under the country grouped design finds no statistically distinguishable difference: XGBoost vs. Random Forest, negative 0.6 percentage points, 95 percent confidence interval negative 3.3 to 2.1; XGBoost vs. the ordinal XGBoost model, negative 1.7 percentage points, 95 percent confidence interval negative 4.4 to 0.1; XGBoost vs. the multinomial logit, positive 0.4 percentage points, 95 percent confidence interval negative 5.7 to 7.3; XGBoost vs. the ordered logit, negative 0.6 percentage points, 95 percent confidence interval negative 5.2 to 4.2. Nested country grouped 5 fold cross validation, in which hyperparameters are re tuned within each fold, confirms this picture: mean accuracy is 0.644, standard deviation 0.069, for XGBoost, 0.648, standard deviation 0.066, for Random Forest, 0.671, standard deviation 0.075, for the support vector machine, 0.674, standard deviation 0.081, for the multinomial logit, 0.676, standard deviation 0.080, for the ordered logit, and 0.637, standard deviation 0.059, for the ordinal XGBoost model, with mean quadratic weighted kappa ranging from 0.689 to 0.720 across models. No classifier's accuracy is decisively higher than the others once the panel structure of the data is accounted for; the modest nominal lead of the support vector machine and the ordered logit models under nested cross validation should not be read as evidence that a linear or kernel model outperforms the tree based ensembles, given the overlapping confidence intervals throughout Table 2. Repeated five by five country grouped cross validation of the two ensemble models gives a mean accuracy of 0.671, standard deviation 0.050, for Random Forest and 0.659, standard deviation 0.053, for XGBoost, consistent with the nested cross validation estimates.

### Confusion matrices under the country grouped design

Figure 3 reports confusion matrices for all six classifiers under the primary, country grouped design. The pattern that emerges is consistent across models: the low and high categories are classified with reasonable accuracy, while the medium category is the hardest to classify for every model, with a substantial share of medium observations misclassified as low. For XGBoost, 235 of 253 low observations and 119 of 259 high observations are classified correctly, but only 140 of 280 medium observations are, with 94 misclassified as low. The same pattern holds for Random Forest, the support vector machine and both logit models, and is only marginally improved by the ordinal XGBoost model, 145 of 280 medium observations correct. Very few observations are misclassified by two categories in either direction, low predicted as high or high predicted as low, so the ordinal structure of the outcome is respected in the sense that errors concentrate between adjacent categories, but this should be read alongside the ordinal metrics in Table 2 rather than as evidence that any model exploits the ordinal structure to a materially greater degree than the others.

**Figure 3:**
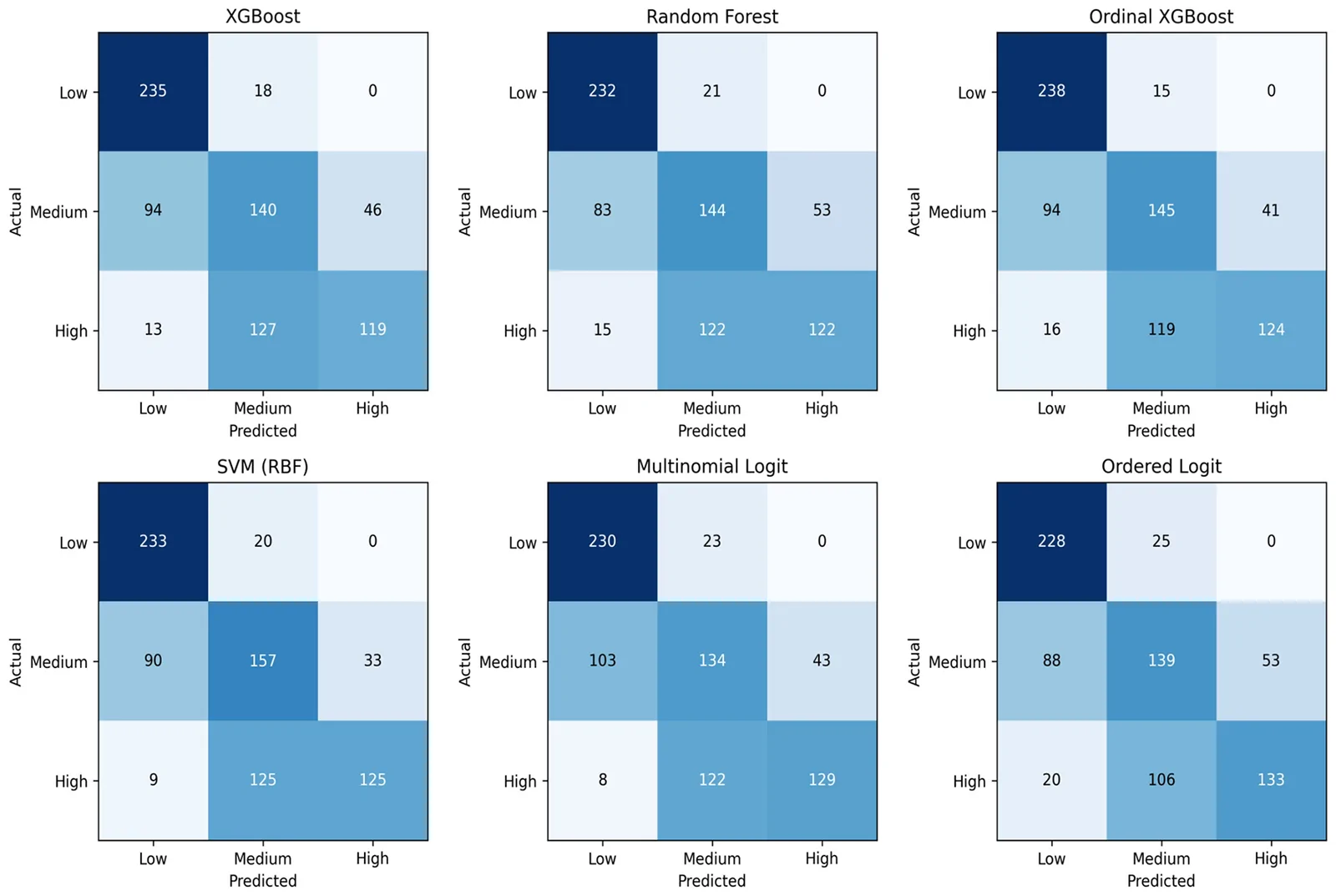
Confusion matrices for six machine learning models under the country grouped validation design. Source: Authors' estimation based on World Development Indicators and OECD data.

### Sensitivity analyses

Table 4 reports sensitivity checks under country grouped 5 fold cross validation for Random Forest and XGBoost, holding the tuned hyperparameters from the primary design fixed except where the analysis itself requires re tuning. The baseline accuracy for this comparison is 0.656 for Random Forest and 0.645 for XGBoost. Replacing pooled tertiles fitted once per split with tertiles computed separately within each calendar year changes accuracy only marginally, 0.648 and 0.652, respectively, indicating the classification is not sensitive to whether the tertile thresholds are allowed to drift across years within the training fold. Replacing sample relative tertiles with fixed, internationally interpretable thresholds of 2.0 and 6.0 tons of CO2 per capita lowers accuracy to 0.614 and 0.603, since these thresholds are not chosen to balance the three classes and, in this sample, produce a noticeably less even split of observations across categories. Excluding the 10 highest emitting countries, Trinidad and Tobago, Brunei Darussalam, Luxembourg, Australia, the United States, Canada, Estonia, the Russian Federation, Kazakhstan and Czechia, raises accuracy to 0.707 and 0.690, consistent with these high emitting, often small or resource intensive economies being harder to classify correctly than the rest of the sample. Restricting the sample to complete cases raises accuracy slightly, to 0.686 and 0.675, while replacing median imputation with iterative, multiple imputation type imputation leaves accuracy essentially unchanged, 0.659 and 0.651, indicating the results are not an artifact of the missing data strategy. Dropping government effectiveness or rule of law individually, given their high pairwise correlation, changes accuracy only marginally in either direction, confirming that neither variable alone is driving the classification once the other remains in the model. Energy use, fossil fuel share and GDP per capita were not available in the underlying dataset for this study and could therefore not be added as an additional robustness check; the authors recommend this as a priority extension once such data become available for the full sample.

**Table 4:** Sensitivity of country grouped classification accuracy to the target construction, sample and predictor set.

Analysis	Random forest accuracy	XGBoost accuracy
Baseline: training fold tertiles, median imputation	0.656	0.645
Year specific tertiles	0.648	0.652
Fixed thresholds, 2.0 and 6.0 t per capita	0.614	0.603
Excluding the 10 highest emitting countries	0.707	0.690
Complete cases only	0.686	0.675
Iterative imputation	0.659	0.651
Without government effectiveness	0.646	0.648
Without rule of law	0.659	0.652

Figures are mean accuracy under country grouped 5 fold cross validation. Hyperparameters are held at the values tuned on the primary, country grouped design except for the two predictor set rows, which are re tuned within each fold because the feature set changes.

### Predictor dependence

Before interpreting the importance rankings, the dependence among the eight predictors was examined directly. Government effectiveness and rule of law are highly correlated, Spearman rho 0.914, with variance inflation factors of 7.77 and 7.75, respectively, the two highest of any predictor and the only ones approaching conventional concern thresholds; the remaining six predictors have variance inflation factors below 1.4. Environmental tax revenue is moderately correlated with both governance variables, Spearman rho 0.48 with government effectiveness and 0.46 with rule of law, and urban population growth is moderately negatively correlated with both, consistent with the development gradient discussed below. Because of this collinearity between the two governance variables, the sensitivity analysis in Table 4 drops each of them in turn; accuracy changes only marginally in either case, indicating that the classification does not depend critically on having both governance variables present simultaneously, though the two are not interchangeable in their SHAP attribution, discussed next.

### Feature importance: permutation importance and SHAP

Figure 4 and Table 5 describe the results for feature importances and SHAP. Given that no classifier shows a decisive accuracy advantage under country grouped validation, the feature importance analysis focuses on the two tree based ensembles, Random Forest and XGBoost, which remain the natural reference models for tree based SHAP attribution. Permutation importance, computed on the country grouped test set with 30 repeats, identifies government effectiveness as the most important predictor for both models, with a mean importance of 0.123 for XGBoost and 0.081 for Random Forest, followed by urban population growth, 0.081 and 0.101, respectively, and environmental tax revenue, 0.039 and 0.033. The remaining five predictors, rule of law, trade openness, gross fixed capital formation, labor force participation and GDP growth, all have permutation importances close to zero for both models, indicating that, conditional on government effectiveness, urban population growth and environmental taxes, these variables add little additional classification accuracy on their own.

**Figure 4:**
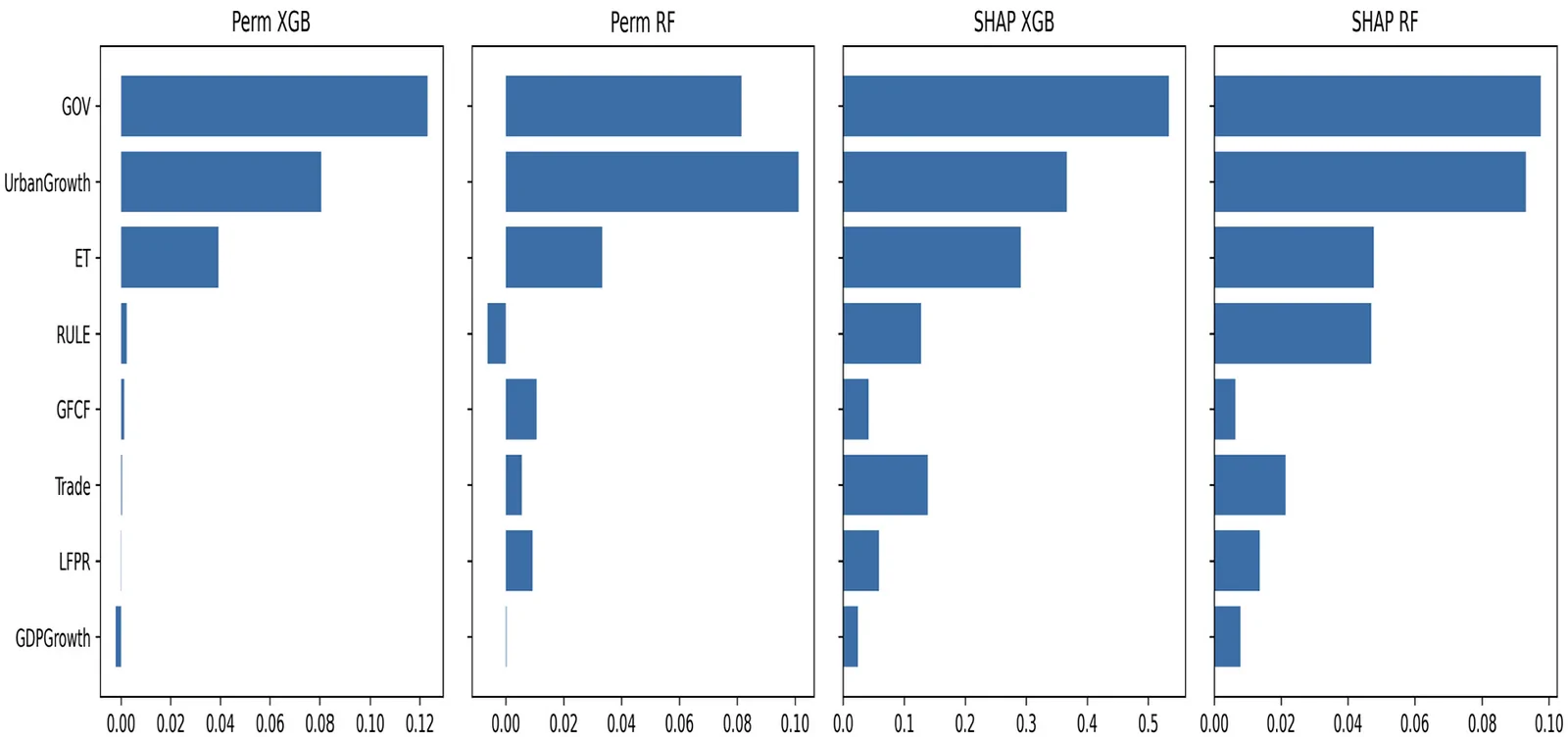
Permutation importance and mean absolute SHAP value for random forest and XGBoost under the country grouped design. Source: Authors' estimation based on World Development Indicators and OECD data.

**Table 5:** Permutation importance and mean absolute SHAP value for random forest and XGBoost, with ranks.

Variable	Perm. XGBoost	Perm. random forest	SHAP XGBoost	SHAP random forest	Rank (mean)
Government effectiveness	0.123	0.081	0.534	0.098	1
Urban population growth	0.081	0.101	0.366	0.093	2
Environmental taxes	0.039	0.033	0.291	0.048	3
Rule of law	0.002	−0.006	0.128	0.047	4
Trade openness	0.000	0.006	0.139	0.021	5
Labor force participation	0.000	0.009	0.059	0.014	6
Gross fixed capital formation	0.001	0.011	0.042	0.006	7
GDP growth	−0.002	0.000	0.024	0.008	8

The SHAP results confirm this ranking. Mean absolute SHAP values place government effectiveness, urban population growth and environmental tax revenue in the top three positions for both Random Forest and XGBoost, with GDP growth the least informative predictor for both. The Spearman rank correlation between the SHAP rankings of Random Forest and XGBoost is 0.952, and the correlation between the permutation and SHAP rankings within XGBoost is 0.881; the correlation between the two models' permutation rankings is somewhat lower, 0.714, reflecting the very small permutation importance, close to the noise floor, of the five lowest ranked predictors rather than any disagreement about the leading three. Recomputing permutation importance separately within each fold of the country grouped 5 fold cross validation, the mean pairwise Spearman rank correlation across folds is 0.555, with a minimum of 0.405, and the same three variables, government effectiveness, urban population growth and environmental tax revenue, occupy the top three positions in three of the 5 folds; the ranking of the remaining five, low importance predictors is noticeably less stable across folds, which should temper any policy weight placed on their precise order. This considerably strengthens the central finding of the study, that institutional quality, urbanization dynamics and environmental tax capacity carry the strongest and most stable predictive signal for classifying countries into CO2 emission levels, while short run GDP growth carries the weakest, but it also cautions against over interpreting the fine grained ranking of the remaining predictors.

### Direction of effects: SHAP summary plots

While the importance rankings identify which variables matter, they do not reveal in which direction they operate. Figure 5 presents SHAP summary plots for the XGBoost model for each of the three CO2 categories. Each dot represents one country year observation in the country grouped test set; its horizontal position measures the contribution of the variable to the classification of the respective category, and its color encodes the value of the variable, red for high values and blue for low values. The plots reveal a coherent development gradient pattern: in the low CO2 panel, low values of government effectiveness, blue dots, push observations toward the low emission category, while high values push away from it; high urban population growth similarly pushes observations toward the low category, consistent with a profile of weak institutions and rapidly growing urban populations typical of developing economies. The high CO2 panel shows broadly the mirror image, with high government effectiveness, high environmental tax revenue and low urban population growth pushing observations toward the high emission category. The higher environmental tax revenue observed among high emission observations should be read as a marker of the fiscal and administrative capacity that accompanies high income, high emission development stages, since countries that collect substantial environmental tax revenue are predominantly high income economies with large per capita emission bases and, consequently, more polluting activity available to tax, rather than as evidence that environmental taxation itself increases emissions. The medium CO2 panel is visibly more compressed than the two extreme panels, with SHAP values clustered near zero for most variables, providing a direct explanation for the weaker recall of the medium class observed throughout Table 2 and Figure 3: the medium category has no distinctive profile of its own and is identified residually, as the absence of the clear signatures that characterize the low and high categories.

**Figure 5:**
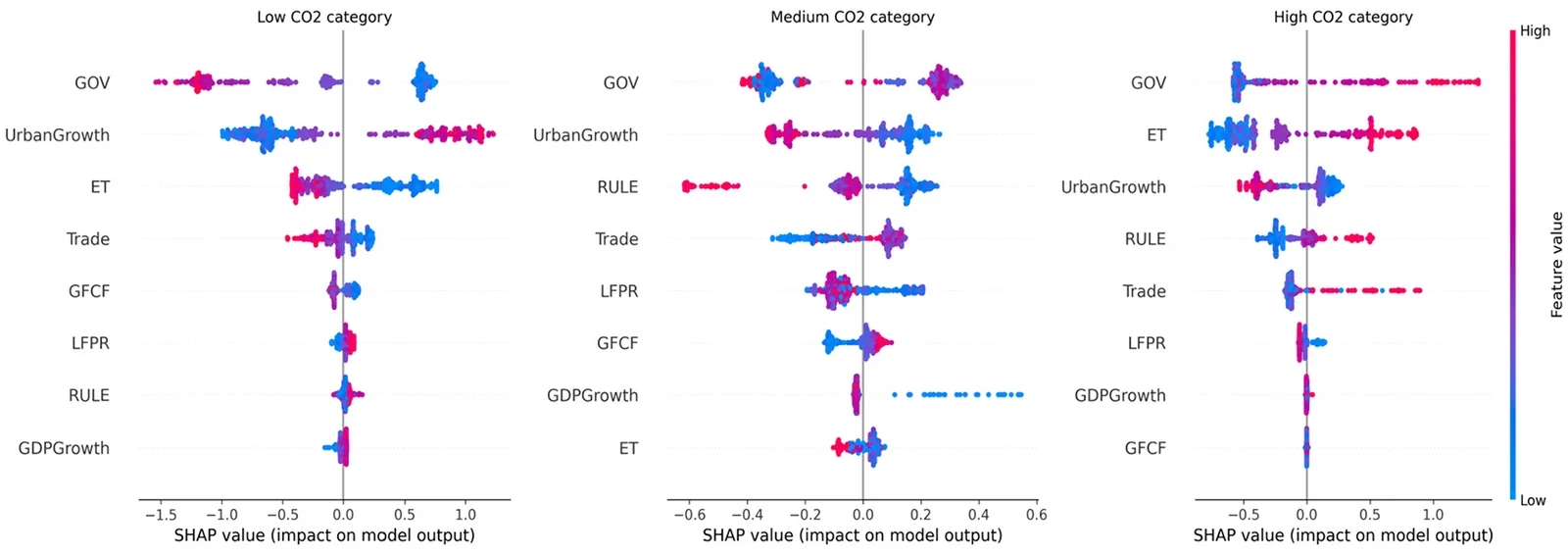
SHAP summary plots illustrating the direction and magnitude of feature contributions across CO2 emission categories, XGBoost model, country grouped design. Source: Authors' estimation based on World Development Indicators and OECD data.

### Accumulated local effects, partial dependence, and SHAP interactions

Figure 6 reports accumulated local effects and centered partial dependence plots for the three top ranked predictors, government effectiveness, urban population growth and environmental tax revenue, and the predicted probability of the low and high categories under the XGBoost model. Both methods agree closely in shape: the probability of the high category rises sharply once government effectiveness exceeds approximately 65–70 on the 0–100 scale, and the probability of the low category falls over the same range; urban population growth above roughly 2–3 percent a year is associated with a sharply higher probability of the low category and a correspondingly lower probability of the high category, while the relationship flattens outside this range; and the probability of the high category rises steeply with environmental tax revenue up to approximately 1.5 percent of GDP and then plateaus, consistent with the fiscal capacity interpretation discussed above rather than a continuously increasing effect.

**Figure 6:**
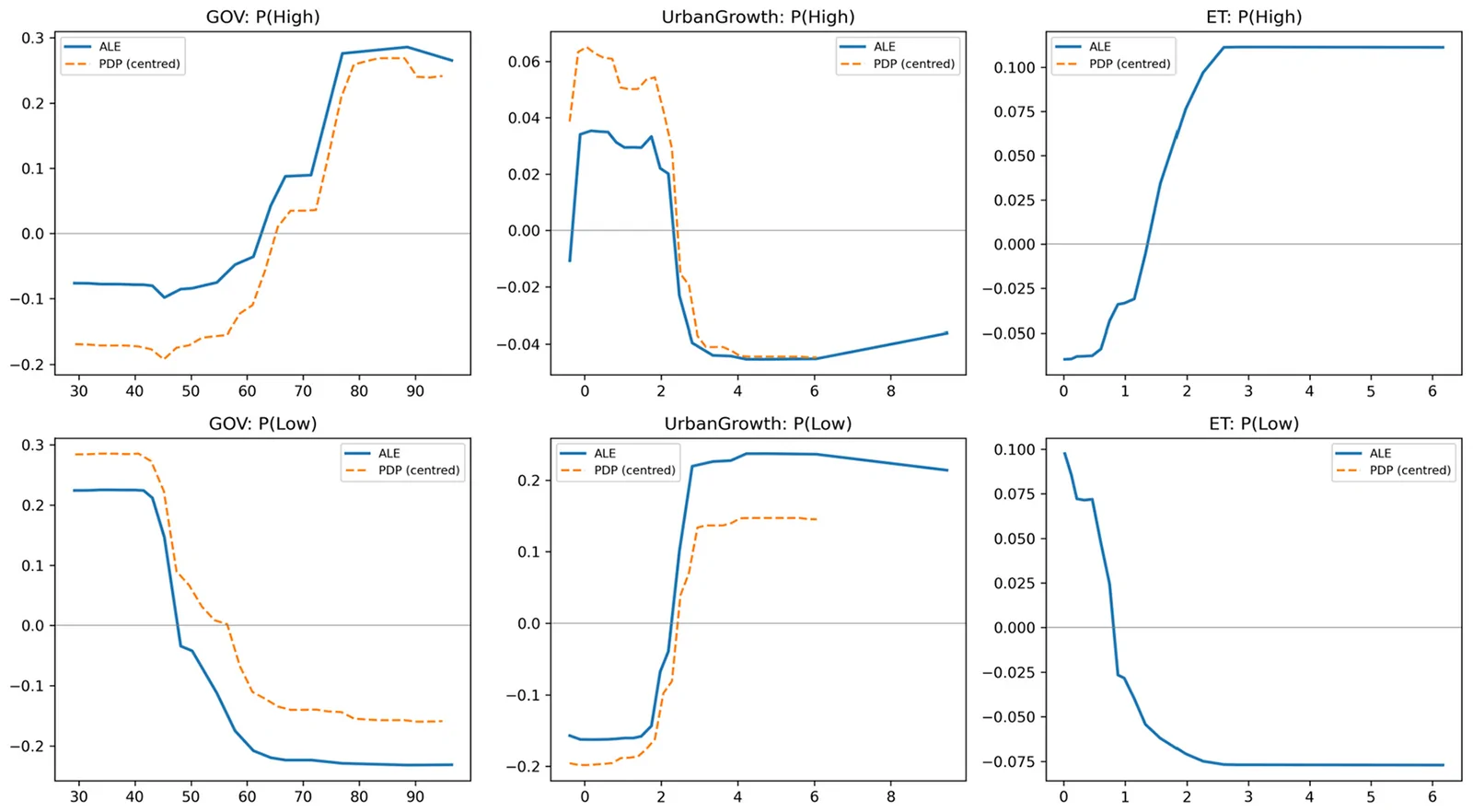
Accumulated local effects and centered partial dependence for the three top ranked predictors, XGBoost model. Source: Authors' estimation based on World Development Indicators and OECD data.

SHAP interaction values for the XGBoost model identify the pairs of predictors that jointly contribute to the classification beyond their individual, marginal effects. The three strongest interactions are between urban population growth and environmental tax revenue, between government effectiveness and urban population growth, and between trade openness and urban population growth, all considerably larger than the interaction between government effectiveness and rule of law, despite the latter pair's high marginal correlation. This indicates that urban population growth conditions how the model uses the other leading predictors, consistent with the SHAP summary plot pattern in which urban population growth and government effectiveness jointly separate the low and high emission profiles, and directly answers the paper's own previously identified direction for future explainability work.

### Local explanations: LIME case studies

To complement the global SHAP analysis with case level evidence, LIME was applied to the XGBoost model under the country grouped design. Rather than a single hand picked observation per class, up to five correctly classified and up to five misclassified observations were drawn at random, with a fixed seed, from each actual emission category in the test set, giving 30 cases in total. Aggregating the absolute LIME weight by feature across all sampled cases confirms the global SHAP ranking at the case level: government effectiveness and urban population growth carry the largest mean absolute weight for both correctly classified and misclassified cases in every category, followed by environmental tax revenue, while GDP growth and gross fixed capital formation contribute the least. Misclassified cases, predominantly medium category observations classified as low or high, show markedly smaller and more evenly spread LIME weights across features than correctly classified cases, consistent with the compressed, non-monotonic SHAP pattern for the medium category identified above: these observations do not carry a strong, single feature signal in either direction, which is itself part of why the model misclassifies them.

### Subgroup performance

Table 6 reports classification accuracy by World Bank region and by OECD membership under the country grouped test set. Performance varies considerably by region: North America, represented by a single country in this test fold, and Sub Saharan Africa, 7 of 33 test countries, show the highest accuracy, 0.958 and 0.923, respectively, while the Middle East and North Africa, represented by 2 test countries, shows the lowest, 0.417, and East Asia and Pacific and Europe and Central Asia show intermediate accuracy around 0.43 to 0.51. Because the country grouped test fold contains as few as one or two countries for some regions, these region level figures should be read as indicative rather than precise, and are reported primarily to flag where classification performance and predictive uncertainty are least reliable rather than to rank regions definitively. OECD member countries are classified somewhat more accurately than non-members, 0.708 against 0.605, consistent with the OECD sourced environmental tax variable being more completely and consistently measured for OECD members, and this gap is discussed further in the Limitations.

**Table 6:** Classification accuracy by world bank region and by OECD membership, country grouped test set, XGBoost model.

Grouping	Group	Countries in test fold	Observations	Accuracy
Region	East Asia and Pacific	6	144	0.431
Region	Europe and Central Asia	8	192	0.505
Region	Latin America and Caribbean	8	192	0.604
Region	Middle East and North Africa	2	48	0.417
Region	North America	1	24	0.958
Region	South Asia	1	24	0.875
Region	Sub Saharan Africa	7	168	0.923
OECD	Non-member	27	648	0.605
OECD	Member	6	144	0.708

Figures for a single validation fold of the country grouped design; small test country counts for some regions mean these estimates should be read as indicative.

## Conclusion and policy recommendations

This paper has analyzed whether trade openness, governance, environmental taxation and a blend of macroeconomic variables can classify countries into low, medium and high CO2 emission levels, and, importantly, whether that classification generalizes to countries and periods the model has not seen. Under a country grouped validation design, in which no country appears in both the training and test sets, classification accuracy for the best performing models is in the region of 0.62 to 0.65, with overlapping confidence intervals across all six classifiers considered, tree based, kernel based and linear alike, and no model achieving a decisive advantage over the others. This is a considerably more modest and more defensible estimate of generalization than the accuracy obtainable under a randomly stratified country year split, which reaches approximately 0.75 to 0.77 with the same tuned pipeline, a gap that directly reflects the persistence of national institutional, trade and urbanization profiles across years rather than genuine predictive skill on unseen countries. Accuracy under a chronological holdout and a genuine 1 year ahead forecasting design, in the region of 0.65 to 0.74, sits between these two figures, indicating that the classifiers generalize better across time within a country than across previously unseen countries.

The feature importance results, confirmed consistently across permutation importance, SHAP and LIME, and stable, though imperfectly so, across cross validation folds, indicate that the most significant and reliable predictors are government effectiveness, urban population growth and environmental tax revenue. Trade openness, rule of law, gross fixed capital formation and labor force participation contribute comparatively little on their own once these three are accounted for, and GDP growth demonstrates the weakest predictive power of all. The SHAP and accumulated local effects analyses additionally reveal the direction of these effects: weak institutions and rapid urban growth characterize low emission country years, whereas strong institutions, low urban population growth and substantial environmental tax collection characterize high emission ones, reflecting a development gradient rather than a set of independent causal levers.

In a policy context, these results are consistent with a role for institutional quality, urbanization management and environmental fiscal capacity in climate monitoring, rather than in short term economic growth indicators, which show little predictive association with a country's emission level. The strong and stable association of governance with the classification suggests governance indicators are useful inputs to climate monitoring systems, though this is a predictive association, not an estimate of a causal effect of governance on emissions. Similarly, the association between environmental tax revenue and the high emission classification most plausibly reflects the fiscal and administrative capacity of high income, high emission economies rather than a causal effect of taxation on emissions, and should not be read as evidence on the effectiveness of environmental tax policy. Translating any of these associations into a specific governance, urbanization or tax policy recommendation would require causal evidence that this predictive design cannot provide; the results are better used to prioritize which countries and which indicators merit closer monitoring and further, causally identified evaluation.

The study has several limitations. First, and most importantly, the models are predictive rather than causal throughout: SHAP, permutation importance and LIME describe how each classifier uses a predictor, not how emissions would respond to a change in that predictor, and this applies with particular force to government effectiveness and environmental tax revenue, both of which are also correlated with unobserved structural characteristics of high income economies. Second, although the tertile cut points are estimated on the training data alone in every reported design, the resulting emission categories remain defined relative to the sampled countries and period rather than to an absolute, internationally fixed threshold; Table 4 shows that year specific tertiles change results only marginally, while fixed absolute thresholds of 2.0 and 6.0 tons per capita lower accuracy for both ensembles, indicating some sensitivity to how the categories are defined. Third, the set of predictors was deliberately restricted to trade, governance, fiscal and macroeconomic variables; direct energy variables such as fossil fuel consumption or the energy mix were not available in the underlying dataset and could not be included, and their inclusion would likely alter the importance ranking reported here, since energy use is a close economic and physical correlate of several of the predictors already included. Fourth, sample composition is constrained by OECD environmental tax data coverage, and classification accuracy is lower for non-OECD members, 0.605, than for OECD members, 0.708, and varies considerably and, for some regions, imprecisely by World Bank region, given how few countries fall in the country grouped test fold for some regions; this coverage constraint should be kept in mind when applying the framework outside the higher income part of the sample. Fifth, the medium emission category remains the hardest to classify under every model and every validation design, consistent with the compressed, non-monotonic SHAP pattern and the LIME evidence for misclassified cases: it appears to be identified residually, as the absence of a distinctive low or high emission profile, rather than through a distinctive feature profile of its own.

Future research can extend this work in several directions. Energy use, fossil fuel share and other direct energy variables should be added once available for the full country sample, to test whether governance, urbanization and environmental taxation retain their leading importance once direct energy variables are available. Additional algorithms, such as neural networks, and a wider hyperparameter search than the one conducted here, could be explored, though the consistency of the results across six already quite different model classes in this study suggests further algorithmic variety is unlikely to change the central finding. Machine learning techniques could also be combined with econometric models, such as fixed or random effects panel models or instrumental variable approaches to institutional quality, to test whether the associations identified here correspond to causal channels, a natural next step given that this study establishes only predictive association. Finally, extending the explainability analysis to a wider set of SHAP interaction pairs, and to a leave one country out validation design as a computationally more expensive but even stricter test of cross country generalization, would further strengthen confidence in the framework's conclusions.

## Data Availability

The raw data supporting the conclusions of this article will be made available by the authors, without undue reservation.
