# Supplementary material for: A global machine learning framework for classifying national CO2 emission levels: responsible and explainable artificial intelligence for sustainable climate governance
Source: Front Artif Intell. 2026 Oct 16;9:1943572. doi: 10.3389/frai.2026.1943572 (PMC13719612; doi:10.3389/frai.2026.1943572)
Supplement: Supplementary file 1 [file Supplementary_file_1.docx]

**Appendix A: List of Countries Included in the Sample**

The table below presents the 135 countries included in the empirical analysis.

| **No.** | **Country** | **No.** | **Country** | **No.** | **Country** |
| --- | --- | --- | --- | --- | --- |
| 1 | Australia | 46 | Belize | 91 | Marshall Islands |
| 2 | Austria | 47 | Bhutan | 92 | Mauritania |
| 3 | Belgium | 48 | Bolivia | 93 | Mauritius |
| 4 | Canada | 49 | Botswana | 94 | Mongolia |
| 5 | Chile | 50 | Brazil | 95 | Morocco |
| 6 | Colombia | 51 | Bulgaria | 96 | Mozambique |
| 7 | Costa Rica | 52 | Burkina Faso | 97 | Namibia |
| 8 | Czechia | 53 | Cabo Verde | 98 | Nauru |
| 9 | Denmark | 54 | Cambodia | 99 | Niger |
| 10 | Estonia | 55 | Chad | 100 | Nigeria |
| 11 | Finland | 56 | Cameroon | 101 | Pakistan |
| 12 | France | 57 | China | 102 | Panama |
| 13 | Germany | 58 | Congo, Rep. | 103 | Papua New Guinea |
| 14 | Greece | 59 | Cote d'Ivoire | 104 | Paraguay |
| 15 | Hungary | 60 | Croatia | 105 | Peru |
| 16 | Iceland | 61 | Cyprus | 106 | Philippines |
| 17 | Ireland | 62 | Dominican Republic | 107 | Romania |
| 18 | Israel | 63 | Ecuador | 108 | Rwanda |
| 19 | Italy | 64 | Egypt, Arab Rep. | 109 | Senegal |
| 20 | Japan | 65 | El Salvador | 110 | Russian Federation |
| 21 | Latvia | 66 | Equatorial Guinea | 111 | North Macedonia |
| 22 | Lithuania | 67 | Eswatini | 112 | Samoa |
| 23 | Luxembourg | 68 | Fiji | 113 | Serbia |
| 24 | Mexico | 69 | Gabon | 114 | Seychelles |
| 25 | Netherlands | 70 | Ghana | 115 | Sierra Leone |
| 26 | New Zealand | 71 | Guatemala | 116 | Singapore |
| 27 | Norway | 72 | Guinea | 117 | Solomon Islands |
| 28 | Poland | 73 | Guyana | 118 | Somalia, Fed. Rep. |
| 29 | Portugal | 74 | Honduras | 119 | South Africa |
| 30 | Slovenia | 75 | Hong Kong SAR, China | 120 | Sri Lanka |
| 31 | Slovak Republic | 76 | India | 121 | Togo |
| 32 | Spain | 77 | Jamaica | 122 | Trinidad and Tobago |
| 33 | Sweden | 78 | Indonesia | 123 | Tunisia |
| 34 | Switzerland | 79 | Kazakhstan | 124 | Uganda |
| 35 | Turkiye | 80 | Kenya | 125 | Ukraine |
| 36 | United Kingdom | 81 | Kyrgyz Republic | 126 | Uruguay |
| 37 | United States | 82 | Lao PDR | 127 | Venezuela, RB |
| 38 | Albania | 83 | Lesotho | 128 | Viet Nam |
| 39 | Antigua and Barbuda | 84 | Liechtenstein | 129 | Zambia |
| 40 | Argentina | 85 | Madagascar | 130 | Korea, Dem. People's Rep. |
| 41 | Armenia | 86 | Malawi | 131 | Bosnia and Herzegovina |
| 42 | Azerbaijan | 87 | Maldives | 132 | Brunei Darussalam |
| 43 | Bangladesh | 88 | Malaysia | 133 | Montenegro |
| 44 | Bahamas, The | 89 | Mali | 134 | Congo, Dem. Rep. |
| 45 | Barbados | 90 | Malta | 135 | St. Lucia |

*Source: Author’s compilation based on the final country-year dataset.*
